# Screening for Lung Cancer Has Limited Effectiveness Globally and Distracts From Much Needed Efforts to Reduce the Critical Worldwide Prevalence of Smoking and Related Morbidity and Mortality

**DOI:** 10.1200/JGO.2017.1700016

**Published:** 2017-10-04

**Authors:** Cherian Verghese, Cristina Redko, Brian Fink

**Affiliations:** **Cherian Verghese** and **Brian Fink**, University of Toledo Medical Center, Toledo; and **Cristina Redko**, Wright State University, Dayton, OH.

## Introduction

Lung cancer is the leading cause of cancer-related mortality worldwide in both men and women. Efforts to reduce lung cancer mortality using chest x-rays (CXRs) for early detection did not show improvements in mortality. More recently, results of the National Lung Screening Trial (NLST), which used low-dose computed tomography (LDCT) scans, appear to improve mortality outcomes. However, LDCT imaging comes at prohibitive costs because of the high number needed to screen as well as inadequate biopsy yields from screen-positive cases. Thus, it is imperative that attempts be made to either improve the efficiency of lung cancer screening or reduce the prevalence of smoking. The latter is especially important considering population increases and the consequently higher prevalence of active smokers. The 2015 WHO report on the global tobacco epidemic highlights that tobacco-related deaths continue to claim more lives than AIDS, malaria, and tuberculosis combined. Hence, continued attempts to reduce the prevalence of smoking are more likely to produce greater mortality reductions than lung cancer screening strategies. Primary preventive strategies have proven benefits but remain underused.

We describe the effectiveness of strategies for smoking control and tobacco-related diseases. We also explain why it is more relevant to increase implementation of these methods than the promotion of screening techniques for lung cancer, especially in low- and middle-income countries.

## Methods

Data were collected after literature review for studies of methods used to reduce smoking prevalence. Information was analyzed in terms of education efforts, effects of increased taxation, and outcomes of media campaigns. Analysis was extended to cost savings from less absenteeism related to smoking-related illnesses, reductions in pregnancy-related complications, increased human productivity from life years preserved, and health care benefits from reduced morbidity. Comparisons were then made with outcomes of the NLST in terms of costs accrued from serial LDCT scans, bronchoscopies, pathology protocols, procedural complications, and absolute improvements in mortality. Incremental costs from individual screenings along with those projected by Medicare over extended periods of screening were analyzed. Last, the potential utility of molecular tumor risk stratification in improving yield of LDCT scanning was assessed.

## Observations

Screening for lung cancer with LDCT appears to improve on results of screening using CXRs. However, two issues need further evaluation: (1) lung cancer is a biologically diverse disease with regard to tumor heterogeneity^1^ and (2) it is not the only way smoking causes morbidity and mortality. Tumor heterogeneity raises questions about biology and which kinds of lung cancers are suitable for early detection and, therefore, have a better chance of cure. In addition, it has become clearer that smoking is directly responsible for many other diseases than lung cancer or obstructive airway disorders.^2^

This is of concern because the global prevalence of smoking is high and worsening in medium- and low-income countries (Table 1).^3^ Rates in the Americas range from 6% in Suriname to 29% in Chile; the average smoking rate is 16% in the United States. There are approximately 48 million active smokers in Latin America and the Caribbean alone. In Russia and France, the prevalence is 33% and 31%, respectively. At 390 million, the Southeast Asia and Oceania regions, however, have the highest concentration of smokers.^4^ All of this has directly increased the worldwide burden of smoking-related illnesses. Carter et al showed that cigarettes accounted for 83% of excess mortality in current smokers, going beyond the 2014 Surgeon General’s report that correlated smoking with excess mortality in 21 disease categories.^2^ The study also revealed associations between smoking and breast cancer, hypertensive heart disease, prostate cancer, intestinal ischemia, and renal failure.

**Table 1:**
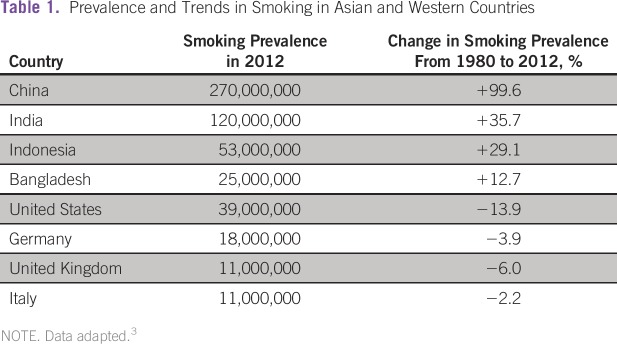
Prevalence and Trends in Smoking in Asian and Western Countries

According to NLST data, screening for lung cancer appears to improve survival, with a 20.3% mortality reduction.^5^ In the study by Aberle et al, 53,454 people were screened between the ages of 55 and 74 years, 26,722 to LDCT scanning and 26,732 to CXRs, with one of either test performed once a year for 3 years, representing T0, T1, and T2 images. Most study subjects (91%) were white, limiting generalizability (Table 2). Study participants were followed for a median of 6.5 years. A total of 1,060 lung cancer cases were detected in the LDCT arm (645 per 100,000 person-years) compared with 941 in the CXR arm (572 per 100,000 person-years). Lung cancer–specific mortality in the LDCT arm was 247 per 100,000, compared with 309 per 100,000 in the CXR group (ie, 2.47 per 1,000 *v* 3.09 per 1,000).Therefore, the number needed to screen to prevent one death from lung cancer is 320 individuals. If the cost of an LDCT scan is $500, the cost will be $480,000 to prevent one death. The cost of an LDCT scan in the NLST conducted between 2002 and 2010 was approximately $285.

**Table 2:**
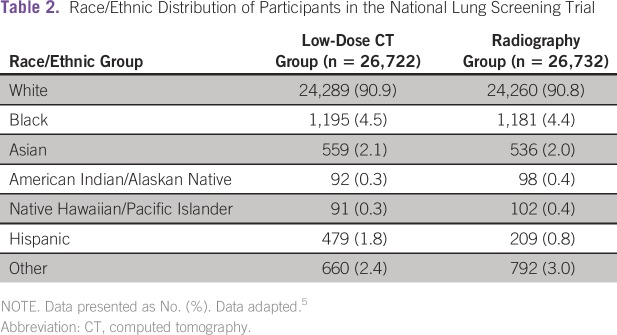
Race/Ethnic Distribution of Participants in the National Lung Screening Trial

These numbers do not take into account the finding that for every 5.4 deaths prevented by LDCT screening, one death was from complications related to the screening itself. Also, 23.3% of tests in the LDCT arm were for false-positive CT findings as compared with 6.5% in the CXR arm. The NLST data have yet to include procedural mortality or costs from procedures and complications resulting from workup for findings that were false positive 94% of the time.

In contrast to LDCT scans, Papanicolaou smears cost only $13 to $66.15 per test, amounting to about $5,392 per life-year saved. Predictive models indicate that implementation of the 2014 hypertension guidelines for US adults between the ages of 35 and 74 years may prevent 56,000 cardiovascular events and 13,000 deaths at < $50,000 per quality-adjusted life year (QALY) gained.^6^ Colonoscopy is also a cost-effective screening modality. However, oncogenesis from smoking is not a simple process and smoking is involved in more disease processes than only cancer. Nearly 8.6 million individuals live with a serious illness caused by smoking and, on average, smokers die more than a decade earlier than nonsmokers.^7^ Currently, one in five deaths is related to smoking, amounting to 443,000 deaths per year in the United States. Worldwide, more than 6 million people die every year from smoking-related illnesses, including lung cancer. Thun et al^8^ looked at 50-year trends in mortality from smoking-related diseases in the United States. Using data from the Cancer Prevention Studies I and II and the US National Health Survey, they showed that death from any cause among active smokers was three times higher than among those who had never smoked. Jha et al^9^ looked at 113,752 individuals ages 25 to 79 years from 1997 to 2004 and confirmed that all-cause mortality was three times higher in current smokers. In fact, smoking cessation at ages 45 to 54 years added nearly 6 years of life compared with those who continued to smoke. And if smoking was stopped by age 40 years, the risk of death was reduced by a significant 90%.

Currently, approximately 3,800 persons younger than 18 years of age start smoking every day and nearly 1,000 become regular smokers.^4^ Worldwide, the burden of new smokers is increasing, and current initiation rates indicate that smoking could be expected to cause the deaths of nearly 1 billion people this century. This raises the question about expenditures incurred by screening when intensifying efforts to reduce smoking by young adults is likely to be more relevant. Contrary to widespread belief, efforts to decrease smoking do work. Although the Hutchinson Smoking Prevention Program may not have shown any difference between study and control populations,^10^ the randomized study by Walter et al^11^ showed that after 6 years of intervention, rates of smoking initiation were significantly lower in schools exposed to educational protocols. A 2005 systematic review in the *Journal of Adolescent Health* did not find any evidence of effectiveness of school-based programs^12^; however, Flay,^13^ in his systematic review of school-based programs, concluded that as long as these programs included at least 15 sessions over multiple years, the social influence model had the potential to reduce smoking by 35% to 40%. Lantz et al^14^ reviewed literature that looked beyond school-based programs at interventions, including the A Stop Smoking in Schools (ASSIST) and Community Intervention Trial for Smoking Cessation (COMMIT) protocols, and recommended that to produce sustained reductions in smoking, it was necessary to combine community programs with policy generation, media interventions, and taxation.

Programs do have implementation expenses, which raises questions about their cost-effectiveness. However, downstream cost savings are substantial (Table 3). Even if initial implementation costs appear to be high, anti-smoking campaigns like those in Massachusetts have saved $3 in health care costs for every $1 spent implementing it.^20^ In another study, Dilley et al^18^ showed that between 2000 and 2009, for every dollar spent by the Washington State Tobacco Prevention and Control program, $5 was saved in health care costs.

**Table 3:**
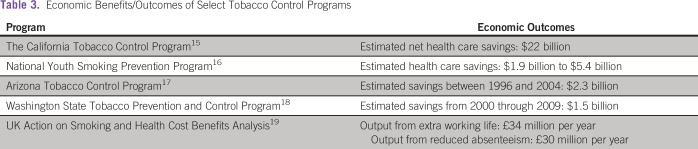
Economic Benefits/Outcomes of Select Tobacco Control Programs

Considering these findings, economic policy experts are of the opinion that with a 5% reduction in smoking rates, states could reduce their health care costs by nearly $2.5 billion per year. These analyses do not take into account the indirect costs of smoking. Data available on the US Centers for Disease Control and Prevention (CDC) website estimate indirect costs, including $156 billion in lost productivity, and $170 billion in health care expenditures.^6^ Pregnancy-related costs are estimated at > $2 billion per year. In children, parental smoking is thought to cause medical problems costing $2.5 billion every year.^21^ Therefore, a person smoking a pack per day at $6.46 per pack costs society an estimated $26.^22^

Recent data from Goodchild et al^23^ show alarming increases in the worldwide economic and social burdens of smoking-related mortality and morbidity. The authors estimate the annual global health care expenditure from smoking-attributable illnesses to be nearly 422 billion (US$) in terms of purchasing-power parity in 2012. This represents nearly 6% of the global health care expenditure. The indirect cost from smoking-related diseases, for which disability is the major factor, remains substantial as well, at $ 1,014 billion. The authors thus estimate the total economic costs of smoking to be $1,436 billion. Part of this cost, unfortunately, comes from the number of life-years lost to smoking-attributable diseases, which comes to nearly 26.8 million years. The adverse effect of this is felt mostly in the labor market from permanent loss of able workers due to early mortality. Developing countries bear 40% of this burden, causing substantial impediment to sustainable development. In 2015, there were 6.4 million deaths worldwide attributable to smoking, and this, interestingly, is a 4.7% increase from 2005.^24^ More importantly, 75% of the related mortality is borne by men, further endangering the well-being of families and communities because, in many developing countries, men are the primary workers. Thus, in developing countries where the tobacco epidemic is still considered by WHO to be at an early stage, it is probably more meaningful to reduce substantially the burden of smoking than it is to screen for lung cancer. This is especially important because 61.7% of the age-standardized disability-adjusted life years are attributable to cardiovascular and respiratory illnesses, with lung cancer accounting for only part of the 20.5% disability-adjusted life years attributable to all cancers caused by smoking.^25^

These social and economic factors make it important to reduce the prevalence of smoking and its burden to society as a whole. It will be difficult to achieve this by implementing a cost-intensive program that must screen 320 individuals to prevent one death due to lung cancer. The costs accrue from expenses related to three LDCT scans each for the 320 needed to screen, in addition to the costs from additional tests, such as positron-emission tomography CT scans (1,868), biopsy procedures (494), bronchoscopies (896), and other surgical procedures, including mediastinoscopy (458).^5^ Data from subgroup analyses by Black et al estimate the cost of the NLST model at $615,000 per QALY gained for former smokers.^26^ The same study nevertheless indicates that screening could be more cost effective if focused on ongoing smokers and individuals in upper risk quintiles. Costs could be < $100,000 per QALY gained in higher-risk subgroups. However, the range for incremental QALYs was considerable (0.0027 to 0.0515) and width of the incremental cost-effectiveness ratio ($32,000 to $615,000 per QALY gained) was large as well. A study by McWilliams et al^27^ looked prospectively at improving the predictability of malignancy in a nodule detected on the first CT scan. They found that nodule size, female sex, age, family history, and upper lobe location increased predictive value. In their Pan-Canadian Early Detection of Lung Cancer Study data set, 700 nodules were detected in 1,871 patients. Of these, 102 were positive for malignancy, amounting to an improved 5.5% yield rate. The Prostate, Lung, Colorectal, and Ovarian Cancer Screening Trial m2012 criteria used additional risk factors, including age, chronic obstructive pulmonary disease, family history, and body mass index, which resulted in an improved detection of 41.3% more lung cancers than what the NLST yielded.^28^ In another subanalyses, Kovalchik et al^29^ divided the NLST population into five quintiles and looked at the incidence of early-stage lung cancer in the different quintiles. Variables included age, body mass index, family history, pack years of smoking, years since smoking cessation, and chronic obstructive pulmonary disease. False-positive results per CT scan–prevented death due to lung cancer decreased from 108 to 78 in the three highest-risk quintiles, and the number needing to be screened to prevent one death changed from 302 to 208 among 60% of participants at highest risk. The yield of LDCT imaging in lung cancer screening could also be augmented through nonclinical methods. Sozzi et al^30^ reported data from the Multicenter Italian Lung Detection trial on the utility of plasma-based microRNA. This reduced the false positivity of LDCT imaging by a factor of five. However, it is questionable as to how much any of these efforts to make lung cancer screening more efficient would improve upon the recent Medicare cost analysis estimating additional costs of LDCT screening at $9.3 billion over 5 years.^31^

Interestingly, efforts were made as early as 2005 to define the cost effectiveness of reducing lung cancer mortality through screening and early detection. Basing their analysis on Markov modeling and using a 2002 price year, Manser et al^32^ suggested that lung cancer screening using low-dose spiral CT was potentially cost effective when calculated for a 27% reduction in mortality against an annual incidence of 552 per 100,000. In their analysis, the incremental cost-effectiveness ratio for men ages 60 to 64 years was $57,325 per life-year saved (in Australian dollars) or $105,090 per QALY saved. They concluded that if $50,000 per life-year saved was used as the measure of cost effectiveness, then reductions in lung cancer mortality would have to be > 20%.^32^ Clearly, costs have since escalated and studies have yet to provide mortality benefits > 20%.

Therefore, alternative measures, including education, taxation, and changing the legal age for smoking from 18 to 21 years, are more likely to have profound cost-effective improvements in morbidity and mortality due to smoking than screening for lung cancer using LDCT scans, especially in developing countries. The effectiveness of behavioral modification, taxation policies, and educational efforts is evident in the recent reductions in the prevalence of smoking. Since 2009, the prevalence has been declining by 0.78% points every year due to factors such as these.^33^ And the CDC estimates that its Tips From Former Smokers program, which educates the public about the harmful effects of tobacco use, may have helped > 400,000 individuals stop smoking and prevented the deaths of nearly 17,000 individuals since its inception in 2012.^33^ Despite this evidence, preventive measures continue to be neglected while screening is being disproportionately promoted. During the fiscal year 2017 in the United States, local governing bodies will collect $26.6 billion from the tobacco settlement. However, they will be spending only about 1.8% of these fund on programs to prevent children from starting smoking and helping adults quit the habit. This is in stark contrast to tobacco companies, which will spend $9.9 billion promoting tobacco products.^34^ This means that for every $1 spent on preventive measures, tobacco companies will outspend the local governments by at least $18. To makes matters worse, even as nearly all states are moving toward implementing lung cancer screening and take advantage of federal funds by doing so, only three of the 50 states in the United States currently fund preventive programs at ≥ 50% of CDC-recommended levels.^35^ Such adverse measures are reflected at the global level as well: WHO data indicate only 37 countries as being on track to achieve the 30% tobacco-reduction target set by the Global Action Plan for prevention and control of noncommunicable diseases from 2013 to 2020. The inadequacy of preventive measures can be seen as a stark contrast to the success of the marketing strategies by tobacco companies—despite a decreasing prevalence of smoking, net population growth has increased the number of cigarettes smoked worldwide to > 6 trillion a year. In fact, smokers in 75 countries continue to consume > 20 cigarettes per person every day. Smoking, therefore, represents a public health issue of grave significance in developing nations, especially considering that the epidemic is still in its early phase in many of these countries.

In conclusion, the findings of the NLST are not precise enough in defining the risk groups in whom screening is cost effective. It is unlikely that the incorporation of other clinical and molecular data will make lung cancer screening with LDCT scans cost effective, especially in developing countries where the smoking epidemic is still in its early stages. It is also necessary to consider the morbidity of smoking and the worldwide burden of cigarette use if actual mortality from smoking is to be reduced. Therefore, it is important to work toward decreasing the prevalence of smoking if the disease burden from smoking is to be reduced. As highlighted in Table 4, educational efforts, limiting access to cigarettes, taxation policies, and legal processes, such as increasing the minimum age to purchase tobacco products, should all be considered in order to reduce the burden of smoking. These strategies are more likely to decrease prevalence and reduce morbidity and mortality from smoking. It is important first to reduce the uptake of smoking by adolescents and young adults through educational measures and, second, to curtail as much as possible the continued use of cigarettes by adults through taxation policies, while also considering raising the legal age for smoking. It is unlikely that the number of labor years lost will be improved by transferring scarce economic resources to lung cancer screening without first reducing the global burden of smoking and all smoking-attributable diseases, particularly in developing countries.

**Table 4:**
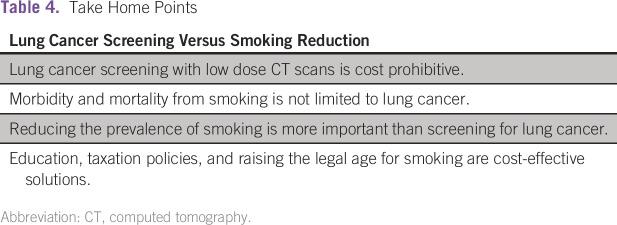
Take Home Points
